# Using Photovoice to Define the Experiences, Needs, Strengths, and Priorities of Gay Men in South Central Appalachia

**DOI:** 10.13023/jah.0701.05

**Published:** 2025-05-01

**Authors:** Ana D. Sucaldito, Lilli Mann-Jackson, Jorge Alonzo, John Chaffin, Scott Rhodes

**Affiliations:** University of North Carolina, Greensboro; Wake Forest School of Medicine; Wake Forest School of Medicine; Western North Carolina AIDS Project; Wake Forest University

**Keywords:** Appalachia, community-based participatory research, LGBTQ+, resilience, photovoice

## Abstract

**Introduction:**

Gay, bisexual, queer, and other men who have sex with men and transgender and nonbinary persons living in rural Appalachia experience profound health disparities. Limited research, however, has focused on the needs, strengths, and priorities of these communities.

**Purpose:**

This study sought to better understand the lived experiences of the vulnerable, underrepresented, and underserved Appalachian gay male community by working with a small group of self-identified gay Appalachian men.

**Methods:**

As part of a larger community-based participatory research study (the *Appalachian Access Project*), four self-identified gay men participated in a photovoice project. Participants took photos based on four group-defined photo assignments that served as discussion triggers to explore experiences and priorities of local LGBTQ+ communities. These discussions were analyzed and member-checked by participants, representatives from community organizations, and academic researchers.

**Results:**

Nine themes emerged. Themes included acknowledgement of the diversity within Appalachian LGBTQ+ community; obstacles faced related to intersectional stigma and discrimination; geographic isolation; community-based peer support to promote belonging, wellbeing, and health; the need for welcoming and safer spaces; peer-to-peer knowledge sharing; self-care strategies; “breaking silences” to raise awareness about underrepresented experiences; and the roles of allyship, advocacy, and activism, to change policies and promote community health.

**Implications:**

This photovoice project sheds light on the needs, strengths, and priorities of gay men living in Appalachia. Those working with these communities could benefit from increasing trust and focus on addressing higher-level environmental factors (e.g., community and policy factors) along with individual and interpersonal factors, to improve health equity for the larger Appalachian LGBTQ+ community.

## INTRODUCTION

Gay, bisexual, queer, and other men who have sex with men and transgender and nonbinary persons (hereafter “gay”) across all races and ethnicities in the United States (U.S.) experience profound health inequities.^1^ These inequities are further exacerbated for those living in the Appalachian Region where access to health care and social services providing health-promoting resources is more difficult, contributing to higher rates of morbidity and mortality.^2^–^3^

There is limited understanding about the health of gay men living in Appalachia from an emic (insider’s) perspective, which is critical for creating efficacious and culturally congruent social services and health interventions to improve their health and wellbeing. Accordingly, this study sought to partner directly with gay men to document their health needs, strengths, and priorities using photovoice.

## METHODS

This project and its parent study (*Appalachian Access Project [AAP]*)^4^ were led by the North Carolina Community Research Partnership, a longstanding CBPR partnership.^2^,^5^

Eligible participants were community health leaders (CHLs) randomized to the delayed-intervention waitlist comparison group of the *AAP* intervention trial.^4^ Participants received $350 for participation; human subjects approval was provided by the Wake Forest University School of Medicine Institutional Review Board.

### Photovoice Sessions and Analysis

**Figure 1:**
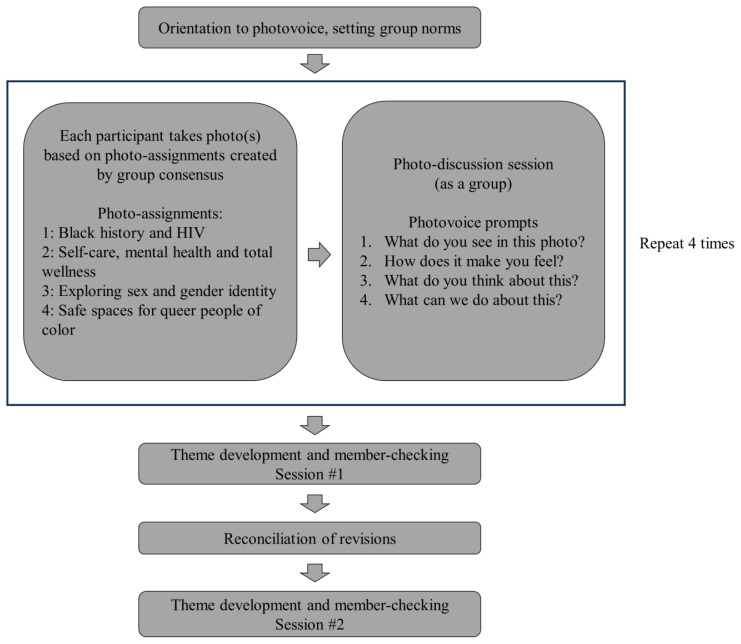
Photovoice Flow Diagram

Photovoice is a qualitative, exploratory method of data collection that produces rich, textured detail about lived experiences.^6^ A figure of data collection and analysis processes, conducted from February – December 2023, is presented in Figure 1.

In brief, participants used their phones to take photos expressing their lived experiences, thoughts, or perspectives guided by group-created photo-assignments. Participants shared one photo at photo-discussion sessions (about 2 – 3 weeks apart). Facilitators used standardized prompts to guide participants from direct observations to more abstract analysis and critical dialog about how images intersected with their experiences as gay men and, largely, those of the LGBTQ+ community living in Appalachia.^6^–^7^

Using constant comparison^8^ and collaboration with photovoice participants, analysts generated initial themes from photo-discussion transcripts. Final themes were created after iterative revisions by photovoice participants, other *AAP* intervention CHLs, members of the study-steering committee, and CBPR partners across two in-person and hybrid meetings (n=11).

## RESULTS

### Participants

Most participants (mean age: 39; range: 30–52 years) self-identified as cisgender gay men (n=3); one as “male genderqueer fluid gay man” (n=1). One participant identified as black/African American and white, one as black/African American and American Indian, one as white, and one as American Indian. One participant withdrew from the project after the first photo-discussion, resulting in three participants who took and discussed twelve unique photographs.

### Themes

The finalized nine themes are detailed below. Quotes supporting each theme are presented in Table 1.

**1. There is great diversity within Appalachian LGBTQ+ communities**. Participants frequently discussed the diversity within the Appalachian LGBTQ+ community. This included, but was not limited to, diversity across sexual orientation, gender identity and expression, race/ethnicity, body size and shape, religion, income, and education.

**2. Intersectional stigma and discrimination are major obstacles to a sense of belonging and to wellbeing and health**. Participants discussed how stigma and discrimination toward those living with one or more marginalized identities (e.g., “living with HIV while gay”) can contribute to isolation from both LGBTQ+ (e.g., gay bars) and non-LGBTQ+ (e.g., clinics providing HIV or other needed services) spaces.

**3. Geographic isolation, compounded by financial barriers, profoundly limits access to network support and other health-promoting resources and strategies**. Participants reported being physically far, and thus also feeling emotionally distant, from potential support systems, such as other LGBTQ+ individuals and communities. They also highlighted how financial barriers worsened this isolation because many do not have the economic stability to access reliable transportation, take time off work, or overcome monetary barriers to entry at community spaces (e.g., the expectation to purchase alcohol or goods).

**4. Community-based peer support is an important strategy to promote belonging, wellbeing, and health**. Participants reiterated a need to connect with others with similar experiences to their own, describing peer support as providing a sense of kinship, belonging, and security in communities where participants did not otherwise feel included or safe.

**5. Welcoming and safer spaces are critical for a sense of belonging and are complex to define**. Characterized by their acceptance and centering of LGBTQ+ identities that are often marginalized, welcoming and safer spaces were reported as critical to fostering a sense of belonging within and outside the LGBTQ+ community. Participants also noted, however, that intersectional identities could impact how truly “safe” these spaces were, as gathering spaces deemed LGBTQ+− friendly by some community members may not be welcoming to LGBTQ+ people of color or, specifically, to transgender persons, due to discrimination from other patrons, the establishment, or larger political context (e.g., policies around bathroom access).

**Figure 2:**
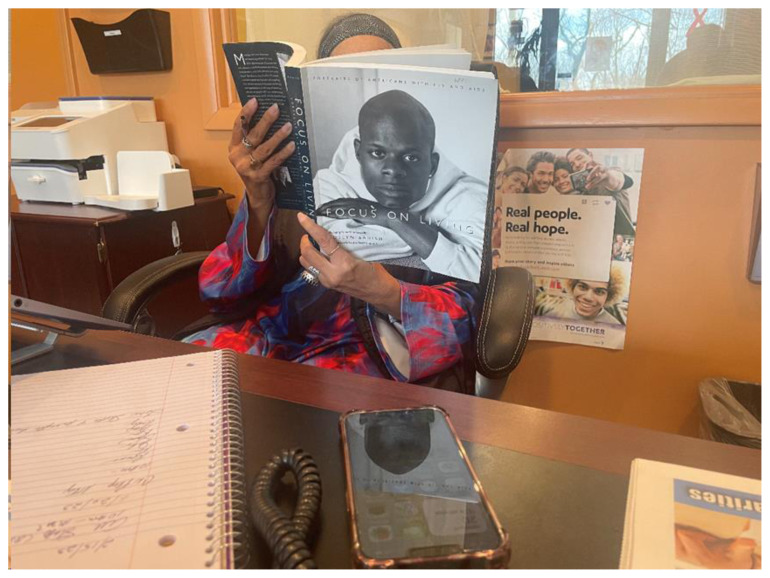
Knowledge-sharing about living with HIV.

**6. There is power in community members sharing knowledge and experiences with one another**. In Figure 2, a photo taken by a participant, an individual reads a book about living with HIV during earlier stages of the epidemic, while sitting in the offices of a local community organization serving those at risk for or living with HIV. Participants described knowledge and experience-sharing like this among the LGBTQ+ community as a method of building, resilience, power, and community.

**7. Intrapersonal resilience is promoted by self-care**. Participants also used self-care (e.g., establishing a daily routine, engaging in self-reflection, and reaching out to peer support networks) to cope with the effects of discrimination based on sexual orientation, gender identity, and race/ethnicity.

**8. There is a need to “break silences” to raise awareness about stigmatized and underrepresented experiences and take action**. Participants underscored the need to bring attention to and address limited representation, understanding, and acceptance for members of the LGBTQ+ community, particularly those who are black, living with HIV, and/or transgender.

**9. Allyship, advocacy, activism, and policy change are needed to promote wellbeing and health of the LGBTQ+ community**. Participants stressed the power that political action had to create safer, more health-promoting environments for LGBTQ+ Appalachian residents, including LGBTQ+ and non-LGBTQ+ people providing individual and community-level support, publicly supporting policies prioritizing LGBTQ+ wellbeing, and organizing for systematic change.

## DISCUSSION

Photos taken by community leaders sparked rich discussions, leading to nine themes regarding the lived experience of being gay in South Central Appalachia. Themes covered a variety of topics but were united in their focus on how the environment (social, physical, and political) affects gay wellbeing and health and the ways that LGBTQ+ Appalachian residents used resilience strategies, particularly external resources, to promote belonging, power, and wellbeing.

Resilience, the ability to overcome and positively respond to negative or harmful stimuli in healthy ways,^9^–^10^ encompasses assets (internal health-promoting factors) and resources (external health-promoting factors). Participant themes acknowledged inequities in Appalachia’s social and built environments (e.g., financial barriers, geographic distance) while heavily emphasizing resources (e.g., social support from peers, knowledge and narrative exchange) working on the broader community and structural levels as ways to promote gay wellbeing and health. These findings aligns with the call to recognize resilience as “scar tissue” from harmful events and shift attention toward addressing societal and systemic levels of harm (e.g., discrimination and inaccessible healthcare systems).^11^ For example, a record 520 anti-LGBTQ+ bills were introduced in state legislatures in 2023,^11^ which may disproportionately affect rural counties such as those in Appalachia and the Southern U.S. with less access to LGBTQ+ health resources.^3^ While individual or interpersonal-level interventions may help mitigate the impacts of such legislation on health, they treat the symptom rather than the cause. Our photovoice themes strengthen the call for decision-makers and public health practitioners and researchers to further focus on social justice and systemic change, and to concentrate efforts on community and environmental levels when designing interventions and working with community organizations.

### Limitations

Despite the initial small sample size and that most participants identified as cisgender men, a larger, more diverse group of LGBTQ+ people living in Appalachia provided input, revisions, and approval of final themes, improving the ability for findings to reflect a broader range of experiences for LGBTQ+ people living in Appalachia. Additionally, data collection was completed prior to damage from Hurricane Helene throughout the region; however, findings may be applicable for efforts to support and address the needs of the LGBTQ+ community in South Central Appalachia as they recover from the long-term impacts of the storm.

## IMPLICATIONS

This study reflects a push to expand the use of resilience to address the larger social, physical, and political environment. While resources and assets created by the LGBTQ+ community should be leveraged and celebrated for their ability to promote wellbeing and health, relying on resilience alone runs the risk of inequitably transferring the burden of healing onto the marginalized, instead of onto the systems creating marginalization. Those working with or on behalf of these communities, such as decision-makers, would benefit from addressing higher-level environmental factors (e.g., policy change, advocacy, and activism), along with individual and interpersonal risk factors, in their efforts to improve health equity for LGBTQ+ Appalachian residents.

**SUMMARY BOX**

**What is already known about this topic?**

LGBTQ+ people experience health inequities across several outcomes, including healthcare access and sexual health outcomes.

**What is added by this report?**

This report details the lived experiences and priorities of gay Appalachian men. Findings include acknowledgement of the diversity within Appalachian LGBTQ+ community; challenges faced related to intersectional stigma and discrimination; geographic isolation; the need for welcoming and “safer” spaces; peer-to-peer knowledge sharing; community-based peer support; self-care strategies; “breaking silences;” and the roles of activism, advocacy, and allyship to change policies and promote community health.

**What are the implications for future research?**

Interventions aimed at improving health equity for the LGBTQ+ community living in Appalachia should address high-level environmental barriers while leveraging the assets and resources built by community members.

## Figures and Tables

**Table 1:** Experiences, Needs, Assets, and Priorities of Gay Men in South Central Appalachia

Themes		Related Quotes
1.	There is great diversity within the Appalachian LGBTQ+ community.	“Each person’s different, — when it comes to the different races, socioeconomics, even different portions of the country – people are so different.” “[This conversation about who may or may not feel safe in a public restroom is] thought-provoking and definitely made us think about how much diversity there is within the community, and different experiences of different people.” “[We should] not put people in boxes but see every person as a human with their own unique experiences and thought processes.”
2.	Intersectional stigma and discrimination are major obstacles to a sense of belonging and to wellbeing and health.	“I don’t know how many times I’ve heard someone say like, ‘I can’t be racist. I’m gay,’ and other things like that. Or as [other participant] was talking about earlier, people can just treat you differently if you’re a person of color and you’re in areas that are mostly white spaces. You get fetishized, you don’t get treated as well as others. You also get looked at more as an object and you’re just not welcome.” “Minorities were dehumanized to begin with, so add a disease [like HIV/AIDS] on top of that. It’s only important when someone rich finally gets it, or someone who’s important or white or someone in a class that’s respected at that time in America. That’s when it becomes a problem.”
3.	Geographic isolation, compounded by financial barriers, profoundly limits access to network support and other health-promoting resources and strategies.	“There are a lot of people out there that don’t have a safer or safe space at all for themselves and might live in an area like in Western North Carolina, where they don’t have access to [a gay bar] or to a community that’s accepting or understanding their experience.” “I try to imagine some of the first spaces that I existed in, and my family. That wasn’t a safe space for me to be my authentic self. The church that I spent a lot of time in during my youth, again, wasn’t a safe space, and as I grew into being a teen and a young adult. There were challenges with, you know, just being allowed to be authentically myself.”
4.	Community-based peer support is an important strategy to promote belonging, wellbeing, and health.	“[In this photo,] I see someone finding a community and not feeling alone. They can help others who are in the same scenario and situation and are feeling alone. And now they have a community that is there for them, and other people who have experiences similar to theirs. It’s just not being alone.”
5.	Welcoming and safer spaces are critical for a sense of belonging and are complex to define.	“A safe space could technically be like, you know, if someone needs to go to the doctor and they find a doctor that’s educated on LGBTQIA+ issues; that’s more of a safe place.” “Honestly, I haven’t really been able to find that many safe spaces. I have a small group of friends, and we do dinner, but there’s a lot of encountering of conflict [in the larger Appalachian community] when the demographic is so different, and everyone looks so different from you.” “Always keep an eye out, have each other’s backs. If you need to guard a bathroom door while someone runs in real fast, just be there for everybody. Try to make the space as safe as you can for everybody in our community.” “One of the things for creating safe spaces, first and foremost as individuals, is doing sort of a reflection of within ourselves. Of how do we make other people feel safe in our presence? And how do we project that into the world? … It’s not as much a physical place that is a safe space. It is how everyone communes in the space actually, that makes things safe.” “That bathroom might be a safe space for me, but for some queer people, it might have a totally different feeling to them.”
6.	There is power in community members sharing knowledge and experiences with one another.	“It hurts me that so many gay people don’t know LGBTQ history and about the civil rights movements for the LGBTQIA+ community. And that a black trans woman threw the first brick and started the whole thing. She was HIV positive; that’s the other aspect of it.” “We all have to keep an eye out for each other, we have to make sure that we’re leaving warnings when necessary, like: ‘This is a safe place for you to go to,’ or ‘I wouldn’t go here.’ ‘This is how I was treated by the regulars here.’ It’s that sense of looking out for each other in a sense of community.”
7.	Intrapersonal resilience is promoted by selfcare.	“Whatever it takes to do that self-care if it’s even if it’s just the smallest thing.” “Doing self-care allows you to come back to the regular grind in everyday life and be able to… see something in a different way. Like that situation that you might have been dealing with for the longest amount of time and just doesn’t seem to change. And then, all of a sudden, you have your moment, and you solve the problem, or you’ve changed the habit.” “The thing about mental health is everybody functions differently so what might work for some people might not for others.” “When you have your basic needs met, it makes it easier for you to get up in the morning after a good night’s rest and be mindful of taking your meds or your vitamins, or whatever it is that you need, drink your water, have your coffee.”
8.	There is a need to “break silences” to raise awareness about stigmatized and underrepresented experiences and take action.	“Silence equals death comes with different types of silences. Some of it is not talking about our history and not talking about things going on with the community.” “I think certain discussions between the generations are not being had, like the sharing of stories about the survival of ourselves and our families in our queer community.” “I think another important thing is to have people be able to tell their stories, instead of telling someone else’s stories. Putting people in roles and positions where you hear directly from them, and not what someone else wrote up about them.”
9.	Allyship, advocacy, activism, and policy change are needed to promote the wellbeing and health of the LGBTQ+ community.	“I think it’s important to visually see people from different groups--the allies--I’m mindful of that.” “Vote, educate other people about legislature, and about how sometimes they’ll use something really big and have a bunch of stuff in the bottom of it--that tiny print that actually affects the LBGTQIA+ community and the trans community.” “I went to an event [for trans youth], and I thought it was very important for them to see a man of color in that role protecting these kids as well. There’s some intersectionality there? And I just think it’s important to be very visually out there, so people can see the support.”
